# Restriction of food intake by PPP1R17-expressing neurons in the DMH

**DOI:** 10.1073/pnas.2100194118

**Published:** 2021-03-22

**Authors:** Caner Caglar, Jeffrey Friedman

**Affiliations:** ^a^HHMI, The Rockefeller University, New York, NY 10065;; ^b^Laboratory of Molecular Genetics, The Rockefeller University, New York, NY 10065

**Keywords:** leptin, metabolism, body weight, food intake, DMH

## Abstract

Leptin-deficient ob/ob mice eat voraciously, and their food intake is markedly reduced by leptin treatment. In order to identify potentially novel sites of leptin action, we used PhosphoTRAP to molecularly profile leptin-responsive neurons in the hypothalamus and brainstem. In addition to identifying several known leptin responsive populations, we found that neurons in the dorsomedial hypothalamus (DMH) of ob/ob mice expressing protein phosphatase 1 regulatory subunit 17 (PPP1R17) constitutively express cFos and that this is suppressed by leptin treatment. Because ob mice are hyperphagic, we hypothesized that activating PPP1R17 neurons would increase food intake. However, chemogenetic activation of PPP1R17 neurons decreased food intake and body weight of ob/ob mice while inhibition of PPP1R17 neurons increased them. Similarly, in a scheduled feeding protocol that elicits increased consumption, mice also ate more when PPP1R17 neurons were inhibited and ate less when they were activated. Finally, we found that pair-feeding of ob mice reduced cFos expression to a similar extent as leptin and that reducing the amount of food available during scheduled feeding in DMH^Ppp1r17^ neurons also decreased cFos in DMH^Ppp1r17^ neurons. Finally, these neurons do not express the leptin receptor, suggesting that the effect of leptin on these neurons is indirect and secondary to reduced food intake. In aggregate, these results show that PPP1R17 neurons in the DMH are activated by increased food intake and in turn restrict intake to limit overconsumption, suggesting that they function to constrain binges of eating.

## Significance

We set out to identify novel neuronal populations controlling feeding behaviors. Therefore, we focused on identifying novel neuronal populations modulating food intake and body weight. In this article, we report that DMH^Ppp1r17^ neurons are activated by increased food intake and that activating them results in decreased food intake and body weight, while inhibiting them leads to increased body weight and food intake. These data suggest that DMH^Ppp1r17^ neurons restrict binges of eating. In addition to its basic science importance, these findings could have therapeutic applications, as they suggest that pharmacologic activation of PPP1R17 neurons could potentially reduce weight in settings of obesity and binge-like eating.

Obesity and its comorbidities, type 2 diabetes mellitus and cardiovascular diseases, are global health problems affecting 400 million people worldwide (1) and causing substantial economic burden (2). Obesity develops as a consequence of positive energy balance, which, in humans, is typically a result of increased food intake. Among mammals, food intake is regulated by an array of sensory and interoceptive signals that regulate the activity of key sets of neurons that control appetite (3).

Leptin is a key metabolic signal that relays information from adipose tissue to the brain to regulate energy balance (4). Leptin-deficient ob/ob mice (also called ob mice) develop extreme hyperphagia and obesity (5), and these abnormalities are corrected by leptin treatment (6). After secretion from adipose tissue, leptin acts primarily on the long isoform of leptin receptor (Leprb) (7), which is expressed in several brain regions, in particular the brainstem and hypothalamus (8–12). Deletion of Leprb in Agouti-related protein (AgRP)-expressing neurons in the hypothalamus leads to increased adiposity similar to that of ob mice, even though other populations also contribute to its effects (13–15).

In this report, we set out to compile an inventory of leptin-responsive neurons by identifying and then testing the function of neurons identified by using PhosphoTRAP, an unbiased profiling method. PhosphoTRAP enables the identification of molecular markers for neurons whose state of activation has changed in response to a defined stimulus (16). We applied PhosphoTRAP to the hypothalamus and brainstem in ob mice before and after leptin treatment, as well as comparing ob to wild-type (WT) mice, and identified a set of previously known leptin-responsive populations. We also found that, as assessed by cFos expression, PPP1R17 neurons in the dorsal medial hypothalamus (DMH) are active in ob mice and suppressed by leptin treatment. Because these neurons are active in ob mice, which are extremely hyperphagic, we initially expected that chemogenetic activation of PPP1R17 neurons in the DMH would increase food intake, while decreasing their activity would diminish it. Surprisingly, we found the opposite effect of PPP1R17 activation on the food intake of ob mice, as it led to decreased food intake, while PPP1R17 inhibition in these animals increased food intake. Similarly, PPP1R17 activation in mice during scheduled feeding, which is also associated with hyperphagia, also decreased food intake and body weight. Finally, we found that pair-feeding of ob mice with a leptin-treated cohort reduced the cFos expression in DMH^Ppp1r17^ neurons, as did limiting the available food during scheduled feeding. We conclude that, rather than being directly regulated by leptin, these neurons are instead activated by hyperphagia and that they act to restrain excessive food intake and limit binges of eating.

## Results

### Identification of Markers for Leptin-Regulated Neurons.

**Fig. 1.**
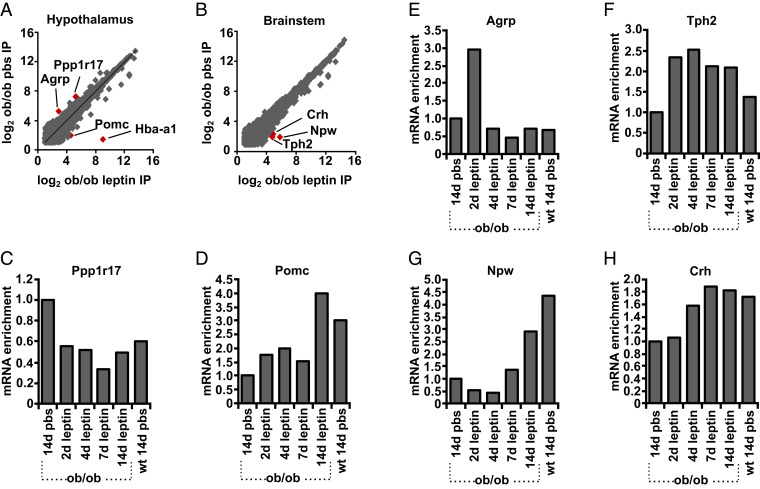
Differential enrichment of genes after leptin treatment. PhosphoTRAP was performed on the samples indicated below, and the RNA abundance determined by the number of RNA-seq reads was plotted in pair-wise comparisons showing (*A*) differential enrichment of genes in pS6 immunoprecipitates determined by RNA-seq in 14 d leptin treatment of ob/ob mice vs. PBS in (*A*) hypothalamus and (*B*) brainstem. TaqMan assays were then performed for the pS6-precipitated polysomes after 14 d PBS treatment of ob/ob mice after leptin treatment (for 2, 4, 7, and 14 d) of ob mice and after 14 d PBS treatment of WT mice. The genes shown are those whose level of enrichment had changed in the comparison between 14 d of leptin vs. PBS (specific genes are shown in *A* and *B*): (*C*) Ppp1r17, (*D*) POMC, (*E*) AGRP, (*F*) Tph2, (*G*) Npw, and (*H*) CRH. Data are expressed as the ratio of fold enrichment (IP per input) for each group of mice divided by the fold enrichment (IP per input) for ob/ob mice treated with PBS for 14 d. Homogenates of pooled hypothalami from 15 to 20 mice are used for each group.

We set out to identify neural populations in the brainstem and hypothalamus that are either activated or inhibited by leptin treatment of ob mice using PhosphoTRAP, an unbiased transcriptomic method to molecularly profile neurons based on a change in activity (16). This method takes advantage of the fact that neuronal activation results in a cascade of signaling events culminating in the phosphorylation of the S6 ribosomal protein (pS6). These phosphorylated ribosomes can then be immunoprecipitated from mouse brain homogenates using a pS6-specific antibody, thereby enriching messenger RNAs (mRNAs) selectively expressed in the activated neuronal population, or depleting RNAs from neurons with reduced activity. After polysome immunoprecipitation and RNA extraction and sequencing, RNAs enriched relative to total RNA have been shown to mark activated neurons, while those that are depleted mark inhibited neurons (16, 17). We applied this method to identify markers for neurons whose state of activation was changed in ob mice relative to WT animals and in ob mice after 14 d of leptin treatment. After polysome precipitation with an anti-pS6 antibody, the number of reads in the immunoprecipitated (IP) RNA for each gene was compared between ob mice treated with leptin for 14 d vs. phosphate-buffered saline (PBS) solution. The enrichment for each gene was calculated as the number of reads in the IP RNA (Fig. 1 *A* and *B*). As expected, we found enrichment for the activity-related gene c-fos in the brainstem and hypothalamic samples from mice treated with leptin (*SI Appendix*, Fig. S1). Consistent with previous studies, we also found enrichment of hemoglobin alpha, adult chain 1 (HBA-A1; Fig. 1*A*). HBA-A1 is expressed in the red blood cells in the brain as a result of constitutively increased mTOR activity in reticulocytes giving rise to enrichment of HBA-A1 mRNA after PhosphoTRAP (17). It is not clear why there was an enrichment of HBA-A1 RNA in the hypothalamic sample but it may reflect increased blood contamination in it.

We next studied the time course of enrichment or depletion of the genes we identified by precipitating polysomes from the brainstem and hypothalamus of ob mice treated with leptin for 2, 4, and 7 d and analyzing the abundance of specific mRNAs using qPCR. Genes that were enriched or depleted in the precipitated polysomes were then validated in histologic analyses of pS6 expression.

### Previously Identified Leptin-Responsive Populations.

**Fig. 2.**
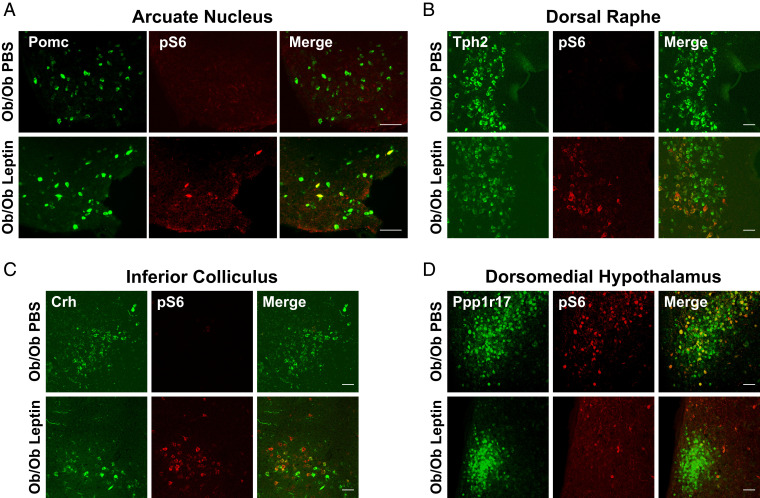
pS6 expression after leptin treatment of ob mice. Dual ISH and IHC in hypothalamus and brainstem was performed for the marker genes and pS6 (244 and 247) as indicated in sections from ob mice treated with leptin (14 d) vs. PBS. (*A*) POMC: ISH for Pomc and IHC for pS6 in arcuate nucleus. Pomc neurons in Arc are activated by 14 d leptin treatment of ob/ob mice. (*B*) Tph2: ISH for Tph2 and IHC for pS6 in dorsal raphe. Tph2 neurons in dorsal raphe are activated by 14 d leptin treatment of ob/ob mice. (*C*) CRH: ISH for Crh and IHC for pS6 in inferior colliculus. Crh neurons in inferior colliculus are activated by 14 d leptin treatment of ob/ob mice. (*D*) Ppp1r17: ISH for Ppp1r17 and IHC for pS6 in DMH. Ppp1r17 neurons in DMH are inhibited by 14 d leptin treatment of ob/ob mice. (Scale bars, 50 μm.)

We found enrichment for several genes previously shown to mark leptin-activated neurons, including the pro-opiomelanocortin (POMC) transcript, which was enriched after leptin treatment (Fig. 1*D*). POMC neurons are located in the arcuate nucleus, and leptin treatment of ob mice is known to increase their activity (18). We further confirmed that leptin treatment increased pS6 in POMC neurons using in situ hybridization (ISH) and immunohistochemistry (IHC; Fig. 2*A*). In contrast, AgRP mRNA was enriched in precipitated polysomes from ob mice relative to WT and depleted after 14 d of leptin treatment, consistent with the fact that AgRP neurons are known to be highly active in ob mice and suppressed by leptin treatment (19) (Fig. 1*E*). We did, however, find that AgRP mRNA was transiently enriched after 2 d leptin treatment while mice were recovering from surgery, presumably in response to a short-term decrease of feeding during the immediate postoperative period (Fig. 1*E*).

We also identified other leptin-regulated neural populations in the brainstem, including neurons expressing tryptophan hydroxylase 2 (TPH2) mRNA, which was significantly enriched after leptin treatment of ob/ob mice (Fig. 1*F*). TPH2 is the rate-limiting enzyme in serotonin production and is primarily expressed in the dorsal raphe nucleus (20). We confirmed that leptin treatment increased pS6 in TPH2-expressing neurons in dorsal raphe histologically by ISH (Fig. 2*B*). RNA-sequencing (RNA-seq) analysis and qPCR results also showed that neuropeptide W (NPW) transcripts were enriched after leptin treatment (Fig. 1*G*). NPW mRNA has previously been shown to be enriched in LepRb neurons in the brainstem (21).

We also identified two populations not previously associated with leptin treatment. RNA-seq analysis followed by qPCR confirmation revealed that corticotrophin-releasing hormone (CRH) transcript is enriched after leptin treatment (Fig. 1*H*). We confirmed the activation of CRH neurons by showing increased pS6 expression in CRH neurons in the inferior colliculus, which is a midbrain nucleus, after leptin treatment of ob mice (note that the midbrain was included in the original brainstem dissection; Fig. 2*C*). While leptin has not been shown to regulate CRH neurons in the inferior colliculus previously, CRH neurons in the hypothalamus are known to reduce of food intake as part of a stress response (22). We also found that the RNA for protein phosphatase 1 regulatory subunit 17 (Ppp1r17) was enriched in the hypothalamus of ob mice relative to WT mice. This neural population has not been previously shown to be leptin-regulated, and we studied it further.

### Functional Studies of Ppp1r17 Neurons in DMH.

The initial RNA-seq analysis and subsequent confirmation using qPCR after polysome precipitation showed that the RNA for Ppp1r17 transcript is significantly enriched in ob hypothalamic samples relative to WT and that this transcript is depleted from polysomes from ob/ob hypothalamus after leptin treatment (Fig. 1*C*). This enrichment was not associated with an altered expression of Ppp1r17 mRNA in the hypothalamus of ob mice relative to leptin-treated ob mice or WT mice, suggesting that the activity of these neurons is increased in ob hypothalamus (*SI Appendix*, Fig. S2). Ppp1r17 is expressed at many sites in the brain, but, in the hypothalamus, it is expressed mainly in the compact region of the DMH (23). Consistent with the data generated using PhosphoTRAP, we found high levels of pS6 immunoreactivity in DMH^Ppp1r17^ neurons at baseline in ob mice and that pS6 expression in these neurons was suppressed by leptin (Fig. 2*D*).

To study the function of the DMH^Ppp1r17^ neurons, we obtained a PPP1R17-cre mouse line, which we confirmed coexpressed cre and Ppp1r17 using ISH for Cre together with IHC for PPP1R17 (*SI Appendix*, Fig. S3*A*). We first evaluated the effect of activating or inhibiting DMH^Ppp1r17^ neurons in WT mice using chemogenetics with activating or inhibitory Dreadds. We injected the cre-inducible viral constructs for inhibition [AAV8-hSyn-DIO-hM4D(Gi)-mCherry] and activation [AAV8-hSyn-DIO-hM3D(Gq)-mCherry] into the DMH of Ppp1r17-Cre mice [50 nL; anteroposterior (AP), −1.80 mm, dorsoventral (DV), −5.1 mm; mediolateral (ML), ±0.3 mm]. Signal transduction from these receptors can be activated by administration of clozapine-N-oxide (CNO), and we compared the effect of CNO to saline in animals expressing the chemogenetic vs. control constructs. Three weeks after stereotactic injections into the DMH, mice were habituated to intraperitoneal (i.p.) injections for 10 d. As expected, after injection of AAV8-hSyn-DIO-hM3D(Gq)-mCherry into PPP1R17-cre mice, c-fos was expressed in mCherry-expressing PPP1R17 neurons 1 h after CNO treatment (*SI Appendix*, Fig. S3*B*). To determine the feeding response following DMH^Ppp1r17^ neurons’ activation, mice were injected i.p. with vehicle or CNO (1 mg/kg) 1 h before the onset of the dark cycle (1900 h).

**Fig. 3.**
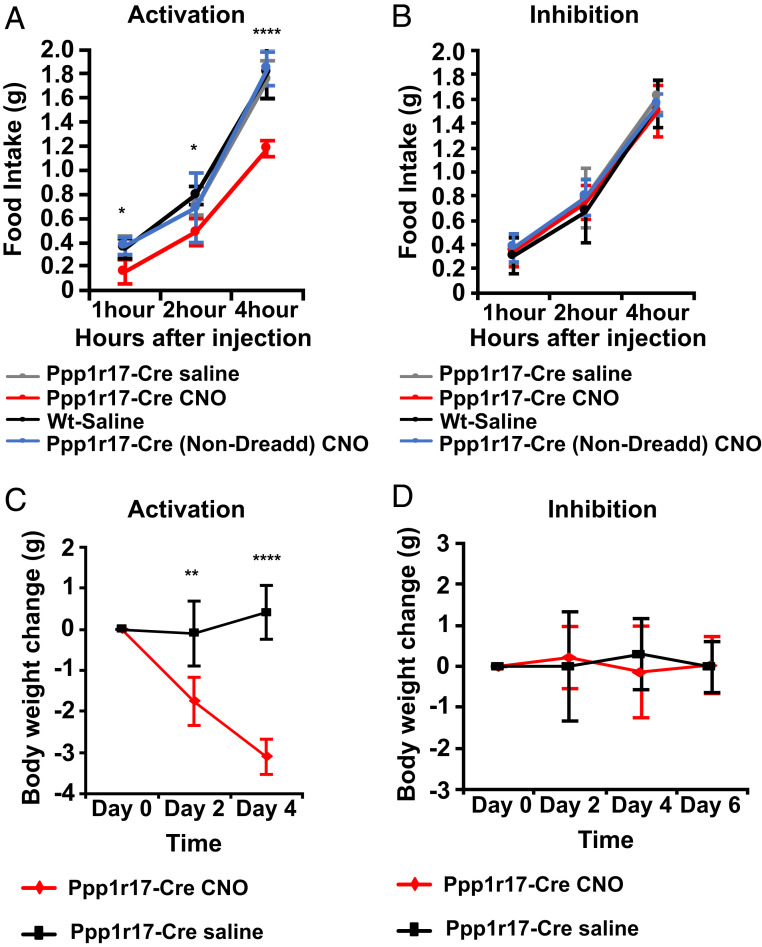
Effect of chemogenetic modulation of DMH^Ppp1r17^ neurons on food intake and body weight in WT mice. (*A*) Chemogenetic activation of DMH^Ppp1r17^ neurons using AAV8-hSyn-DIO-hM3D(Gq)-mCherry. DREADD-induced activation of DMH^Ppp1r17^ neurons significantly decreases food intake over the course of 4 h. (*B*) Chemogenetic inhibition of DMH^Ppp1r17^ neurons using AAV8-hSyn-DIO-hM4D(Gi)-mCherry. DREADD-induced inhibition of DMH^Ppp1r17^ neurons does not affect food intake over the course of 4 h. (*C*) Body weight of Ppp1r17-Cre mice is significantly reduced after chronic activation of DMH^Ppp1r17^ neurons by i.p. injection of CNO two times per day. (*D*) Body weight of Ppp1r17-Cre mice does not significantly change after chronic inhibition of DMH^Ppp1r17^ neurons by i.p. injection of CNO two times per day (*P < 0.05, **P < 0.01, ***P < 0.001, and ****P < 0.0001, two-tailed unpaired *t* test; *n* = 5 to 6 mice). All error bars are mean ± SD.

Chemogenetic activation of DMH^Ppp1r17^ neurons in WT mice by CNO acutely decreased food intake during the dark cycle relative to the vehicle control, with ∼33% decrease in consumption at 4 h (*P* < 0.0001; Fig. 3*A*). Chronic activation of these neurons was associated with significant weight loss after 2 and 4 d of treatment (*P* < 0.01 and *P* < 0.0001, respectively; Fig. 3*C*). The PhosphoTRAP data and histologic analyses of pS6 in these neurons suggested that these neurons are not active in WT mice, and, consistent with this, acute inhibition of DMH^Ppp1r17^ neurons by CNO did not show an effect on food intake relative to the vehicle control (Fig. 3*B*). Similarly, chronic inhibition of DMH^Ppp1r17^ neurons by CNO did not show a significant effect on body weight relative to the vehicle control at 6 d of treatment (Fig. 3*D*). As an additional control, we injected CNO into animals that received injections of a control virus expressing GFP in Ppp1r17 neurons in the DMH and failed to observe a change in food intake (Fig. 3 *A* and *B*).

### Functional Studies of DMH^Ppp1r17^ Neurons in ob Mice.

**Fig. 4.**
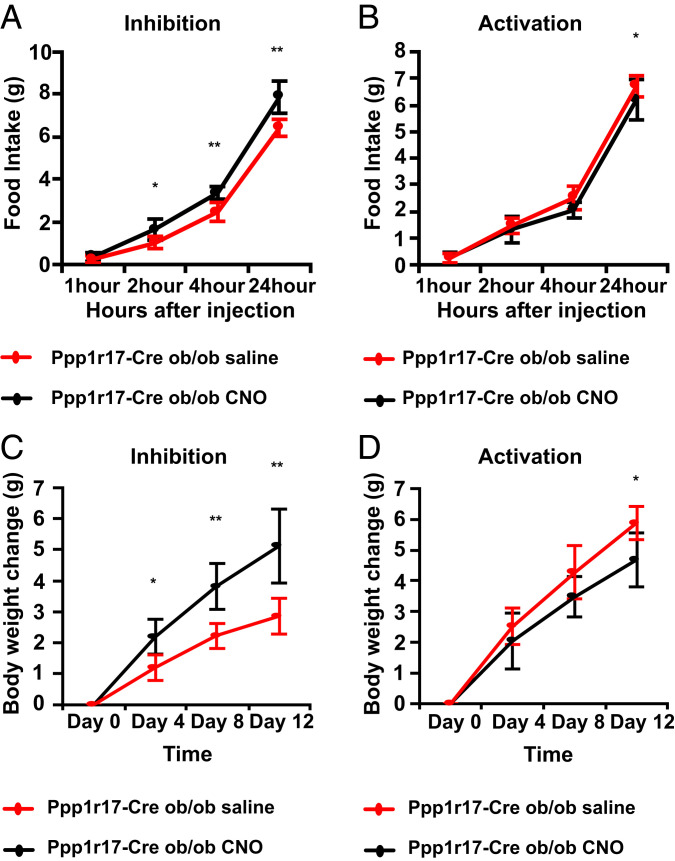
Effect of chemogenetic modulation of DMH^Ppp1r17^ neurons on food intake and body weight in ob/ob mice. (*A*) Chemogenetic inhibition of DMH^Ppp1r17^ neurons in ob/ob mice. DREADD-induced inhibition of DMH^Ppp1r17^ neurons significantly increases food intake over the course of 24 h. (*B*) Chemogenetic activation of DMH^Ppp1r17^ neurons in ob/ob mice. DREADD-induced activation of DMH^Ppp1r17^ neurons significantly decreases food intake over the course of 24 h. (*C*) Body weights of ob/ob mice are significantly increased after chronic inhibition of DMH^Ppp1r17^ neurons by i.p. injection of CNO two times per day. (*D*) Body weights of ob/ob mice are significantly reduced after chronic activation of DMH^Ppp1r17^ neurons by i.p. injection of CNO two times per day at the end of 12 d (*P < 0.05, **P < 0.01, ***P < 0.001, ****P < 0.0001, two-tailed unpaired *t* test; *n* = 5 mice). All error bars are mean ± SD.

We hypothesized that inhibition of Ppp1r17 neurons did not affect food intake in WT mice because they show low activity at baseline in fed WT animals. In contrast, DMH^Ppp1r17^ neurons are active in ob/ob mice (Fig. 2*D*). We thus tested whether inhibition of DMH^Ppp1r17^ neurons would modulate food intake and body weight in ob/ob mice. To investigate this, we crossed ob/ob mice to Ppp1r17-cre mice (Ppp1r17-Cre::Ob/Ob) followed by injection of the inhibitory AAV8-hSyn-DIO-hM4D(Gi)-mCherry or activating AAV8-hSyn-DIO-hM3D(Gq)-mCherry Dreadds into the DMH. Inhibition of DMH^Ppp1r17^ neurons in ob mice with CNO significantly increased acute food intake by 62% at 2 h (*P* < 0.05), 36% at 4 h (*P* < 0.01), and 22% at 24 h (*P* < 0.01; Fig. 4*A*). Similarly, chronic inhibition of Ppp1r17 neurons in ob mice with daily injections of CNO resulted in a significant and progressive increase of body weight after 4, 8, and 12 d of treatment relative to saline (*P* < 0.05, *P* < 0.01, and *P* < 0.01, respectively; Fig. 4*C*). In contrast, activation of DMH^Ppp1r17^ neurons after CNO injections into mice that received the activating AAV8-hSyn-DIO-hM3D(Gq)-mCherry Dreadd showed a small but significant decrease of food intake of 8% at 24 h (*P* < 0.05; Fig. 4*B*) that was also associated with decreased body weight at day 12 (*P* < 0.05; Fig. 4*D*). Overall, these results are consistent with the results seen after modulation of Ppp1r17 neurons in WT mice and show that DMH^Ppp1r17^ neurons can bidirectionally regulate food intake and body weight in ob/ob animals. As described below, we next evaluated the function of Ppp1r17 neurons in mice during scheduled feeding, a protocol that, similar to ob mice, is associated with an increase of food intake during a short time interval (i.e., a binge of eating).

### Functional Analysis of DMH^Ppp1r17^ Neurons During Scheduled Feeding.

**Fig. 5.**
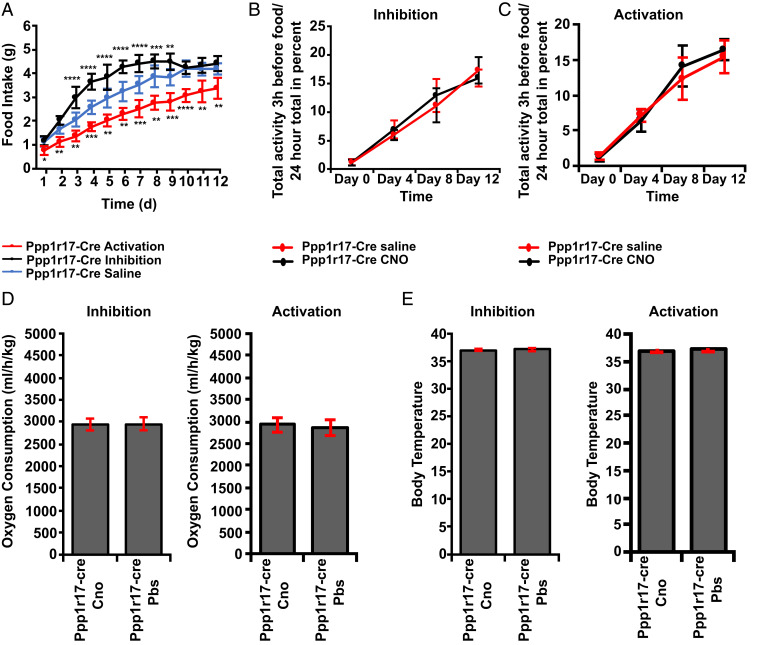
Effect of chemogenetic modulation of DMH^Ppp1r17^ neurons on food intake, body weight, and FAA during scheduled feeding. (*A*) DREADD-induced activation (treatment *P* < 0.0001) and inhibition (treatment *P* < 0.0001) of DMH^Ppp1r17^ neurons significantly alters food intake during scheduled feeding after i.p. injection of CNO 4 h before food presentation. Two-way repeated-measures ANOVA comparing treated and control groups. (*B*) DREADD-induced inhibition of DMH^Ppp1r17^ neurons does not affect FAA. (*C*) DREADD-induced activation of DMH^Ppp1r17^ neurons does not affect FAA. (*D*) DREADD-induced activation or inhibition does not alter oxygen consumption during scheduled feeding. Two-tailed unpaired *t* test comparing treated (Ppp1r17-cre CNO) and control group (Ppp1r17-cre PBS; *E*). DREADD-induced activation or inhibition does not alter body temperature during scheduled feeding. Two-tailed unpaired *t* test comparing treated (Ppp1r17-cre CNO) and control group (Ppp1r17-cre PBS; *n* = 8 mice; *P < 0.05, **P < 0.01, ***P < 0.001, and ****P < 0.0001). All error bars are mean ± SD.

The DMH has been shown to play a prominent role in mediating the biologic response to scheduled feeding (24). In a scheduled feeding paradigm, animals are provided with food during a 3-h window during the light phase rather than being fed ad libitum during the dark phase. After a 1- to 2-wk period during which the animals learn the new feeding schedule, animals then show a marked increase in the rate of food intake, now consuming their total daily intake during the 3-h window (Fig. 5*A*). In addition, the mice show a marked increase in locomotor activity just prior to the onset of this window, known as food anticipatory activity (FAA), as well as other physiologic responses (Fig. 5 *B* and *C*).

To test the role of DMH^Ppp1r17^ neurons in the adaptation to scheduled feeding, we injected the inhibitory AAV8-hSyn-DIO-hM4D(Gi)-mCherry or the activating AAV8-hSyn-DIO-hM3D(Gq)-mCherry into the DMH of different groups of Ppp1r17-cre mice. Each day for 12 d, CNO (1 mg/kg) or vehicle was injected i.p. 4 h prior to providing food (i.e., at 8:00 during the light phase). Inhibition of DMH^Ppp1r17^ neurons during scheduled feeding by CNO led to a rapid and highly significant increase of between 16 and 44% of daily food intake relative to the vehicle control between day 3 and day 9 during the 3-h feeding window (*P* < 0.0001; Fig. 5*A*), while activation of DMH^Ppp1r17^ neurons by CNO led to a significant decrease of between 19 and 33% daily food intake between day 1 and day 12 (*P* < 0.0001; Fig. 5*A*). Scheduled feeding is generally associated with weight loss until the animals learn that they need to consume their food during the 3-h window. In these studies, where we modulated the activity of these neurons before the animals had regained their weight, inhibition of DMH^Ppp1r17^ neurons led to a more rapid regain of body weight of 3% by 12 d (*P* < 0.01), while activation of DMH^Ppp1r17^ neurons resulted in 4% slower weight regain, with animals not returning to the weight of controls until 12 d (*P* < 0.001; *SI Appendix*, Fig. S4). Modulating the activity of DMH^Ppp1r17^ neurons did not show any effect on any of the other physiologic responses to scheduled feeding, including FAA (Fig. 5 *B* and *C*), oxygen consumption (Fig. 5*D*), or core temperature (Fig. 5*E*).

### Ppp1r17 Activity Is Regulated by Increased Food Consumption, Not by Leptin.

**Fig. 6.**
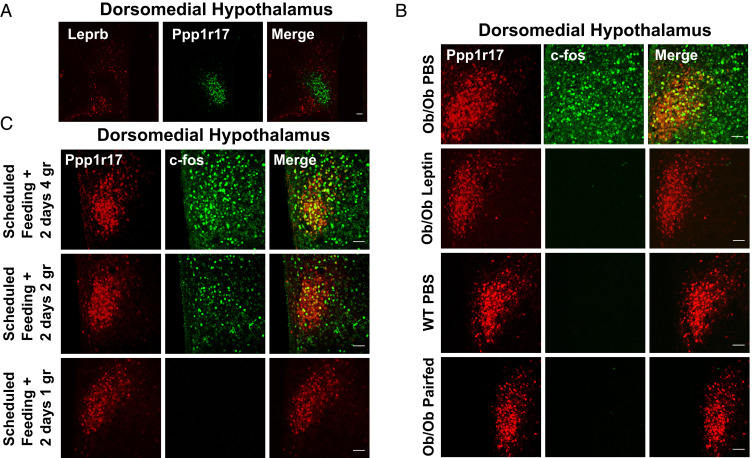
cFos expression in Ppp1r17 neurons in ob mice and during scheduled feeding. (*A*) IHC for Ppp1r17 and TdTomato in ObRb TdTomato mice. DMH^Ppp1r17^ neurons do not express Leprb. (*B*) cFos expression in DMH^Ppp1r17^ neurons in ob/ob mice is reduced by 14 d pair-feeding. (*C*) cFos expression in DMH^Ppp1r17^ neurons in scheduled feeding during the 3-h feeding window in response to different amounts of chow. Food is provided to mice between Circadian Time (CT)4 and CT7: (*Upper*) 4 g of food, the amount normally consumed during the 3-h feeding window; (*Middle*) 2 g of food, the amount mice consume in 3 h after a fast; and (*Lower*) 1 g, the amount consumed during the dark phase within 3 h. (Scale bars, 50 μm.)

Leptin treatment of ob mice reduces food intake, and we had initially expected that inhibiting the activity of a leptin-activated population such as the DMH^Ppp1r17^ neurons would also reduce food intake. However, as shown above, we instead found that inhibiting DMH^Ppp1r17^ neurons increased food intake in both ob mice and during scheduled feeding, while activating them decreased food intake in these settings as well as in WT mice. These findings thus appeared paradoxical, leading us to consider the possibility that these neurons may not, in fact, be directly regulated by leptin. Consistent with an indirect effect of leptin on these neurons, IHC revealed that DMH^Ppp1r17^ neurons do not express the leptin receptor (Fig. 6*A*). Fluorescent dual IHC results showed that LepRb neurons in DMH do not colocalize with PPP1R17 neurons, and, rather, that there is a separate population of LepRb neurons, by using LepRb-Ires-Cre::tdTOM mice that express the tdTomato fluorescent protein specifically in LepRb cells (Fig. 6*A*).

To further assess whether these neurons are directly regulated by leptin, we compared cFos expression in DMH^Ppp1r17^ neurons in leptin-treated ob mice to ob mice pair-fed with the leptin-treated group. DMH^Ppp1r17^ neurons in ob mice showed high levels of cFos expression, and pair-feeding led to a similar decrease in cFos and pS6 expression, as did leptin treatment (Figs. 2*D* and 6*B* and *SI Appendix*, Fig. S5). In aggregate, these data show that the activity of these neurons, assessed using cFos and pS6 expression, is increased during binges of eating and diminished by a reduction of food consumption. Thus, the data further suggest that the effect of leptin is secondary to its appetite-suppressing effect.

To further address this, we next analyzed the effect of reducing food intake during scheduled feeding on cFos expression in animals after they had fully acclimated to this protocol. In a standard scheduled feeding study, by day 12, animals consume their total daily intake (∼4 g of food) during the 3-h window. We generated three cohorts of mice who had fully acclimated to the new feeding schedule, and then tested the effect of limiting the food that was available during this same 3-h window over the course of several days. Group 1 continued to receive 4 g, the amount they had been consuming ad libitum, during the 3-h feeding period. Group 2 received 2 g during the 3-h window (still an increased rate of consumption), and group 3 received 1 g of food, which is similar to the normal rate of consumption of WT mice during the dark phase within 3 h. Consistent with the possibility that these neurons are activated by significantly increased food consumption, we found that, while DMH^Ppp1r17^ neurons in group 1 and 2 continued to express cFos, cFos was no longer induced in DMH^Ppp1r17^ neurons in group 3 (Fig. 6*C*). These results showed that DMH^Ppp1r17^ neurons are activated during periods of intense, supraphysiologic binges of food intake.

## Discussion

Leptin has pleiotropic effects on a number of behaviors and physiologic responses, including food intake, sexual activity, locomotion, glucose metabolism, the reward value of food intake, reproductive capacity, and others (8–12). While numerous distinct neural populations have been shown to regulate one or more of these responses, it is unclear to what extent these known populations account for all of leptin’s effects (8–12). We addressed this using PhosphoTRAP (16) to create an inventory of leptin-responsive neurons and potentially identify additional neural populations in the brainstem and hypothalamus that mediate some portion of leptin’s effects. In addition, because PhosphoTRAP identifies any neurons whose activity has changed after leptin treatment, it can also identify indirect (i.e., downstream) targets of leptin action.

Several neuronal populations were identified after leptin treatment of ob mice, including neurons in the compact DMH-expressing PPP1R17, an inhibitor of protein phosphatase 1 (23, 25, 26). PPP1R17 neurons are more active in ob mice relative to WT mice, and their activity is suppressed by leptin treatment. However, this reduced activity is also observed during pair feeding which, together with the finding that PPP1R17 neurons do not express LepRb, indicates that leptin’s effects on these neurons are indirect and secondary to its appetite-suppressing effects. Consistent with this, the activity of these neurons is increased during periods of intense hyperphagia after scheduled feeding and reduced when the available food is limited.

These neurons act to limit the amount of food that is consumed during periods of marked overconsumption as shown by the effect of chemogenetic inhibition to increase the food intake of ob mice. This effect is opposite to what would be expected for a leptin-suppressed population. Similarly, inhibiting PPP1R17 neurons also increased the food intake and body weight of mice during scheduled feeding, another circumstance during which mice consume greatly increased amounts of nutrient. In contrast, activating PPP1R17 neurons in ob mice, in chow-fed WT mice, and during scheduled feeding reduced food intake and body weight. In aggregate, these data suggest that DMH^Ppp1r17^ neurons are activated in response to increased food consumption, i.e., a binge of eating, and in turn reduced food intake. The finding that DMH^Ppp1r17^ neural inhibition further increased the food intake and weight of ob mice indicates that, even when leptin is absent, there are neural pathways that nonetheless act to restrain hyperphagia. Ppp1r17 inhibits protein phosphatase 1, whose substrates include the S6 ribosomal protein (26). Since PhosphoTRAP depends on the correlation of S6 phosphorylation and neural activity, the identification of these neurons could have been a result of this catalytic activity rather than an effect of changing neural activity. However, we confirmed that the enrichment of Ppp1r17 is indeed a result of a change of neural activity by also showing increased cFos in this DMH population in ob mice and that leptin treatment of ob mice reduces cFos expression.

Numerous sensory and interoceptive signals regulate food intake and body weight in addition to leptin, including a set of endocrine and neural satiety signals from the gastrointestinal tract (27). These signals, including CCK, GLP1, and others, are processed largely by specific populations of neurons in the nodose ganglion, area postrema, and nucleus of the solitary tract (NTS) and relayed to other feeding centers (28). Recent studies have also shown that vagal inputs to the nodose ganglion and NTS can also reduce feeding. For example, neurons expressing GPR65 and projecting into NTS regulate the entry of food into the intestine (29). Furthermore, vagal gut afferents have been shown to regulate meal size via a negative-feedback mechanism and have been implicated in brain reward (30). Our finding that Ppp1r17 neurons are activated when food intake is acutely increased and act to reduce appetite raises the possibility that they are downstream targets of the brainstem centers that process these gut-derived signals. Indeed, the characteristics of DMH^Ppp1r17^, which are activated by feeding and decrease food intake, resemble those of several populations of vagal afferent neurons projecting to NTS, including those expressing CCK1R, GLP1R, VGLUT2, and GPR65 (29). Consistent with this possibility, it has been shown that several regions in the brainstem, including NTS, parabrachial nucleus, and periaqueductal gray matter, innervate the DMH (31). In addition, leucine-sensing NTS neurons also regulate food intake by sending projections to the DMH (32). Several brain regions in the hypothalamus also innervate the DMH, including preoptic (POA), paraventricular, and arcuate nuclei (31). For example, GAD2 neurons in the POA regulate body temperature by sending projections into DMH neurons innervating the rostral raphe pallidus (33). AgRP neurons in the arcuate nucleus also innervate the DMH and control heart rate (34). Based on this, we hypothesize that DMH^Ppp1r17^ neurons are an indirect downstream target of vagal afferent neurons projecting into the NTS, although there may also be additional functional inputs from other hypothalamic regions and elsewhere. Studies defining the neural inputs and outputs of DMH^Ppp1r17^ neurons and their functions can be performed to address this.

In addition to regulating food intake during scheduled feeding (16), the DMH plays an important role to regulate the hyperactivity of mice just prior to animals receiving food during a scheduled feeding paradigm. Specific lesions in the DMH region also blunt the increase in body temperature, locomotor activity, and wakefulness that are observed in unlesioned mice just before food presentation during scheduled feeding (24) Leprb neurons in anterior DMH also modulate body temperature and locomotor activity, while Leprb neurons in posterior DMH regulate food intake (35). However, the modulation of the activity of the PPP1R17 neurons does not alter any of these other responses and appears to be limited to an effect on food intake.

While we did not identify many novel populations of leptin-responsive neurons, these studies also further validate the utility of PhosphoTRAP. These included neurons expressing AgRP (36–39) and POMC in the hypothalamus (36, 38, 40) in the arcuate nucleus. We also found enrichment of NPW mRNA in the brainstem. Previous studies of the transcriptome of brainstem identified a subpopulation of LepRb neurons also expressing NPW, though the effect of leptin was not evaluated (21). NPW mRNA is also expressed in the dorsal raphe nucleus, periaqueductal gray matter, and ventral tegmental area, and our data suggest that leptin regulates their activity (41, 42). We also found that activity of TPH2 neurons is regulated by leptin. While some studies have suggested that these neurons are not leptin-responsive, recent studies have suggested that TPH2 neurons in dorsal raphe can regulate the reward value of food (43). Our data are consistent with an effect of leptin on these neurons, though our results do not establish whether this is direct or indirect. We also found that the activity of CRH neurons in the inferior colliculus is regulated by leptin. While this neural population has not previously been shown to be leptin-responsive, similar to leptin, CRH suppresses food intake (44, 45). These data raise the possibility that CRH neurons in inferior colliculus could play a role in mediating some of the effects of leptin. We also found an apparent enrichment of HBA-A1 mRNA in the leptin-treated samples, suggesting increased reticulocyte contamination (17). The basis for this is not clear, and it could be a result of increased blood contamination in some of the samples. Alternatively, this could reflect differences in cerebral blood flow in obese vs. lean animals, as changes in the cerebral artery structure have been observed with obesity (46). Further studies will be required to address these or other possibilities.

However, some populations of known leptin-responsive neurons were not identified, including those expressing SF1 and neurotensin (47, 48). The basis for this is not clear, though it is possible that increased S6 phosphorylation is not durable in these neurons over longer periods of time. Since we did not perform PhosphoTRAP experiments after acute leptin treatment, we may have missed leptin target neurons that are only activated acutely. In addition, some SF1 and neurotensin neurons in the hypothalamus do not express LepRb (47, 48), and it is possible that leptin could elicit different effects on different subpopulations (i.e., some could be activated while others might be inhibited, canceling out an observable effect). Thus, while these results suggest the possibility that most of the leptin-responsive neurons in the hypothalamus and brainstem have already been identified, we cannot exclude the possibility that, similar to SF1 and neurotensin neurons, additional novel populations of leptin responsive neurons were missed.

In summary, we identified a neural population in DMH that controls food intake and body weight as part of a counter-regulatory response to markedly increased food consumption. These neurons normally limit the food intake and body weight of ob mice, as well as mice who binge-eat during scheduled feeding, revealing that there are neural mechanisms in hyperphagic mice that, in fact, restrict their intake. Furthermore, the finding that DMH^Ppp1r17^ neurons are activated during periods of intense food intake and in turn limit consumption raises the possibility that defects in this response lead to binge eating and could thus be of clinical relevance.

## Materials and Methods

### Animal Treatment.

All experiments were approved by The Rockefeller University Institutional Animal Care and Use Committees and performed following the National Institutes of Health guidelines. Male mice were used for behavioral studies (>8 wk). Mice were housed in a 12-h light/dark cycle with ad libitum access to water and food except as otherwise specified in text.

Obrb-cre [B6.129(Cg)-Leprtm2(cre)Rck/J; Jackson Laboratory stock no. 008320] was used to express cre in lerpb neurons. Ppp1r17-cre [Tg(Ppp1r17-cre) NL146Gsat/Mmucd] mice were used to express cre in PPP1R17 neurons. ROSA-loxSTOPlox-tdTomato [Jackson Laboratories stock no. 007909; B6.Cg-Gt(ROSA)26Sortm9(CAG-tdTomato)Hze/J] was used to express the reporter gene in the presence of Cre recombinase by using lox-STOP-lox sequence.

### Viral Vectors.

All viruses used in these studies were obtained from Addgene. AAV8-hSyn-DIO-mCherry (control virus), AAV8-hSyn-DIO-hM3D(Gq)-mCherry (activation), or AAV8-hSyn-DIO-hM4D(Gi)-mCherry (inhibition) were used for chemogenetic studies.

### Stereotaxic Surgery.

Mice aged 8 to 12 wk were anesthetized by using 2% isoflurane. Paxinos mouse brain atlas was used to identify coordinates. PPP1R17-Cre mice were injected with 50 nL AAV8-hSyn-DIO-mCherry (control virus) or AAV8-hSyn-DIO-hM3D(Gq)-mCherry (activation) or AAV8-hSyn-DIO-hM4D(Gi)-mCherry (inhibition) virus in the DMH bilaterally (coordinates: AP, −1.80 mm; DV, −5.1 mm; ML, ±0.3). All listed DV coordinates are relative to bregma. At 10 min after injection, the needle is slowly retracted. Suturing or surgical clips are used to close the skin.

### IHC.

After anesthetizing mice with isoflurane, mice were transcardially perfused with PBS followed by 10% formalin. Dissected brains were postfixed in 10% formalin at 4 °C overnight. Brain slices at 40 to 50 μm were obtained by using a vibratome. Free-floating sections were washed with PBS three times, and incubation was performed in blocking buffer (PBS, 0.1% Triton-X, 2% goat serum, 3% bovine serum albumin [BSA]) for 1 h at room temperature to block sections. Next, sections were incubated at 4 °C overnight with primary antibodies in block solution. After overnight incubation, PBS and 0.1% Triton-X was used to wash sections three times for 15 min, followed by incubation in Alexa Fluor-conjugated secondary antibodies at 1:1,000 dilution at room temperature for 2 h. Last, sections were washed in three times in PBS and 0.1% Triton-X for 15 min and mounted on slides. Secondary antibodies were Alexa Fluor-conjugated (Life Technologies). All images were captured using confocal microscopy (Zeiss or Leica).

Primary antibodies used were rabbit anti-PPP1R17 (1:300; Sigma-Aldrich HPA047819), rabbit anti–c-fos (1:1,000, Santa Cruz sc52), goat anti–c-fos (1:1,000; Santa Cruz sc52G), phospho-S6 Ser244, Ser247 (1:1,000; Thermo Fisher scientific no. 44-923G), and chicken anti-GFP (1:1,000; Abcam ab13970).

### RNA-Seq and TaqMan Array Analysis.

TopHat and Cufflinks apps were used to analyze RNA-seq results. *Mus musculus* assembly mm10 was used to annotate alignments. Genes that have more than two fragments per kilobase of transcript per million mapped reads are depicted in the RNA-seq graph. TaqMan probes were used to validate RNA-seq results. A QuantiTect Reverse Transcription Kit was used to obtain complementary DNA (cDNA). TaqMan Gene Expression Master Mix was used to quantify the abundance of the genes. Transcript abundance was normalized to rpL27. Differential fold enrichment values were calculated by ΔΔCt method. *P* value was calculated by unpaired two-tailed *t* test.

### Awake Chemogenetic Studies to Measure Thermogenesis.

Mice that were injected with DREADD [hM3D(Gq) or hM4D(Gi)] or control virus into DMH were injected with 1 mg/kg CNO or 0.9% saline and single-housed. Core body temperature was measured in the home cage during the animal’s light phase by using an anal probe (Braintree Scientific). *P* value was calculated by unpaired two-tailed *t* test.

### Chemogenetic Studies Using Metabolic Home Cages.

Mice that were injected with DREADD [hM3D(Gq), hM4D(Gi)] or control virus into DMH were placed in metabolic cages and single-housed for 1 wk to allow them to habituate to social isolation. Metabolic parameters were recorded automatically in metabolic cages with an automated home cage phenotyping system (TSE-Systems). Locomotor activity and oxygen consumption of mice were monitored during metabolic phenotyping. Custom software for metabolic cages analyzes the beam breaks to record locomotor activity. Oxygen consumption is measured by an indirect gas calorimetry module that is a part of TSE-Systems. After habituation, locomotor activity is recorded for 12 d during scheduled feeding. CNO is injected intraperitoneally 4 h before the food presence for 12 d. Oxygen consumption is recorded during light phase of mice 1 h after CNO injection for 3 h. All data were collected and processed with PhenoMaster software. For food intake and body weight, *P* value was calculated by two-way repeated-measures ANOVA testing. For FAA and oxygen consumption, *P* value was calculated by unpaired two-tailed *t* test.

### Food Intake and Body Weight Assay for Chemogenetic Studies.

Mice were single-housed and placed in home cages during food intake assay. For acute food intake measurement, CNO was injected intraperitoneally just before the dark phase, and measurements of food intake were performed at 1, 2, and 4 h post i.p. injection of CNO for WT mice experiments and 1, 2, 4, and 24 h after i.p. injection of CNO for ob mice experiments. For body weight and food intake measurement at 24 h, CNO was injected twice per day at a concentration of 1 mg/kg. *P* value was calculated by unpaired two-tailed *t* test.

### Animals: Diet and Leptin Normalization.

WT and ob/ob C57BL/6J mice were purchased from Jackson Laboratories. Microosmotic pumps (model 2002; Durect) were used to dispense either leptin (150 ng/h in PBS) or vehicle (PBS). Pumps were implanted to 10-wk-old mice subcutaneously, and mice were single-housed. Recombinant murine leptin was purchased from Amylin Pharmaceuticals.

### Fluorescent ISH with IHC.

Anti-sense digoxigenin-labeled riboprobe were synthesized for cre (953 bp), ppp1r17 (958 bp), pomc (915 bp), tph2 (631 bp), and crh (934 bp). To quench endogenous peroxidase activity, 40 μm free-floating brain sections were treated with 3% H_2_O_2_ for 1 h at room temperature, followed by incubation in 0.20% acetic anhydride for 30 min, and incubated in 1% Triton-X for 30 min each. For prehybridization, free-floating brain slices were incubated in hybridization buffer at 62 °C (50% formamide, 5× saline-sodium citrate (SSC), 5× Denhardt’s solution, 250 μg/mL baker’s yeast RNA, 500 μg/mL single-stranded DNA) for 1 h. Hybridization is carried out at 62 °C with riboprobe overnight. Free-floating slices were washed once in 5× SSC and two times with 0.2× SSC at 62 °C. Slices were incubated in the primary antibody with riboprobe. Sections were washed with 0.2× SSC and buffer B1 (0.1 M Tris, pH 7.5, 0.15 M NaCl) briefly followed by blocking in TNB (1% blocking reagent in B1; Roche no. 1096176) for 1 h at room temperature. Anti–digoxigenin-POD antibody (1:100; Roche no. 11207733910) was carried out overnight at 4 °C. To combine IHC with FISH, sections are incubated with a secondary antibody conjugated to Alexa 488/594 for 1 h at room temperature before riboprobe was detected. TSA Plus Fluorescence System (Perkin-Elmer NEL744) was used to develop riboprobe according to the manufacturer’s instructions.

### PhosphoTRAP Experiment.

After loading 150 μL protein A Dynabeads (Invitrogen 10002D) with 4 µg of Phospho-S6 Ser244, Ser247 (Thermo Fisher Scientific 44-923G) in buffer A (150 mM KCl, 10 mM Hepes, 1% Nonidet P-40, 5 mM MgCl_2_, and 0.05% IgG-free BSA), beads loaded with pS6 antibody were washed in buffer A three times. Following euthanasia by cervical dislocation, 15 to 20 hypothalamus and brainstem samples were dissected in buffer B containing 4 mM NaHCO_3_, 1× HBSS, 100 µg/mL cycloheximide, 2.5 mM Hepes, and 35 mM glucose on ice and pooled separately. Hypothalami and brainstems are transferred to separate glass homogenizers (Kimble Kontes 20). Buffer C (protease and phosphatase inhibitor mixes, 150 mM KCl, 10 mM Hepes, pH 7.4, 100 nM calyculin A, 2 mM DTT, 100 U/mL RNasin, 5 mM MgCl_2_, 100 µg/mL cycloheximide) was used to resuspend hypothalami and brainstems, and a speed homogenizer (Glas-Col) was used to homogenize samples at 4 °C. Homogenized samples were transferred to Eppendorf tubes and centrifuged at 2,000 × *g* for 10 min at 4 °C to clarify samples. The supernatant was transferred into a new Eppendorf tube containing 90 µL of 1,2-diheptanoyl-sn-glycero-3-phosphocholine (Avanti Polar Lipids; 100 mg/0.69 mL) and 90 µL of 10% Nonidet P-40. After mixing the solution, it was centrifuged at 17,000 × *g* at 4 °C for 10 min. A total of 20 μL of the supernatant is collected in another tube containing 350 μL of buffer RLT (RNeasy Micro kit; Qiagen no. 74004) and kept at −80 °C for purification as input RNA. Remaining supernatant was collected into a new Eppendorf tube and kept on ice for 10 min for immunoprecipitation. Buffer D (10 mM Hepes, pH 7.4, 350 mM KCl, 5 mM MgCl_2_, 2 mM DTT, 1% Nonidet P-40, 100 U/mL RNasin, and 100 µg/mL cycloheximide) was used to wash the beads four times. After the second wash, beads were collected in a new tube and incubated on ice for 10 min, followed by a final wash. A total of 350 µL of buffer RLT was added into the tube containing beads to elute RNA. Magnets were used to separate beads from RNA. To perform RNA-seq analysis, cDNA was obtained by using the SMARTer Ultralow Input RNA for Illumina Sequencing Kit and then sequenced by using an Illumina HiSEq 2000 system.

## Supplementary Material

Supplementary File

## Data Availability

All study data are included in the article and/or supporting information.
