# Supplementary material for: Restriction of food intake by PPP1R17-expressing neurons in the DMH
Source: Proc Natl Acad Sci U S A. 2021 Mar 22;118(13):e2100194118. doi: 10.1073/pnas.2100194118 (PMC8020659; doi:10.1073/pnas.2100194118)
Supplement: Supplementary File [file pnas.2100194118.sapp.pdf]

Leptin

Phospho-trap

Pomc
Tph2
Crh
Npw

Agrp

Ppp1r17

DMH Ppp1r17

-Ob/ob mice
-During scheduled feeding

-Leptin treatment
-Pair feeding
-Decreased food intake during scheduled feeding

Chemogenetic Activation

CNO

hM3D(Gq)

Food intake

Body weight

FAA

O_2 cons.

Body temp.

Chemogenetic Inhibition

CNO

hM4D(Gi)

Food intake

Body weight

FAA

O_2 cons.

Body temp.

Schematic Drawing of Main Findings (created with biorender.com)

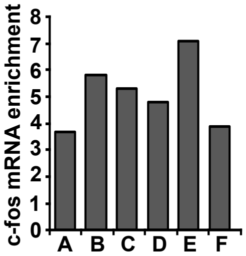

Figure S1. c-fos mRNA enrichment in pS6 immunoprecipitates determined by taqman A: ob/ob mice treated with PBS for 14 days B: ob/ob mice treated with leptin for 2 days C: ob/ob mice treated with leptin for 4 days D: ob/ob mice treated with leptin for 7 days E: ob/ob mice treated with leptin for 14 days F: Wt mice treated with Pbs for 14 daysData are expressed as the ratio of fold enrichment(IP/input) for each group of mice.

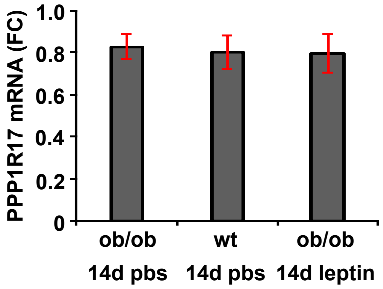

Figure S2. Ppp1r17 mRNA expression in hypothalamus is determined by Taqman assays. ΔΔCT analysis is used to calculate the fold change of Ppp1r17 mRNA expression in hypothalamus of ob mice treated with leptin for 14 days, ob mice treated with PBS for 14 days and Wt mice treated with PBS 14 days. n=3 mice. All error bars are mean ±SD

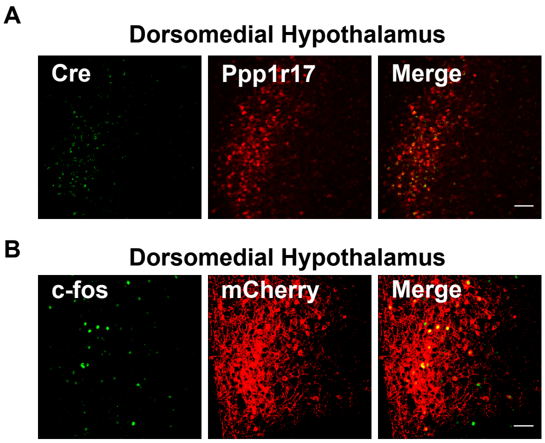

Figure S3. Validation of Ppp1r17-cre mice A. Cre is expressed in $DMH^{Ppp1r17}$ neurons of Ppp1r17-Cre mice. B. IHC for c-fos and mCherry in DMH. AAV8-hSyn-DIO-hM3D(Gq)-mCherry is injected into DMH of Ppp1r17-cre mice. 1 hour after i.p. CNO injection, c-fos is detected in mCherry expressing neurons in DMH of Ppp1r17-cre mice.

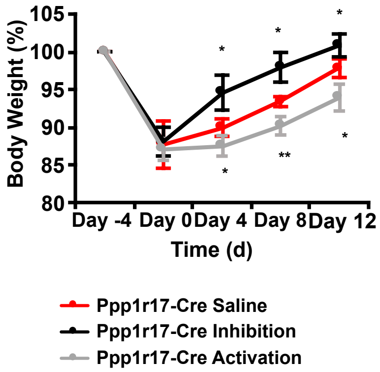

Figure S4. Body weight changes after activating and inhibiting $DMH^{Ppp1r17}$ neurons during scheduled feeding. DREADD induced activation (Treatment: p-value<0.001) and inhibition (Treatment: p-value<0.01) of $DMH^{Ppp1r17}$ neurons significantly alter food intake during scheduled feeding by i.p injection of CNO 4 hours before food presentation. Two-way RM ANOVA comparing treated and control groups n=5 mice. Scheduled feeding starts at Day 0. * $p < 0.05$, ** $p < 0.01$, *** $p < 0.001$, and **** $p < 0.0001$ All error bars are mean ±SD

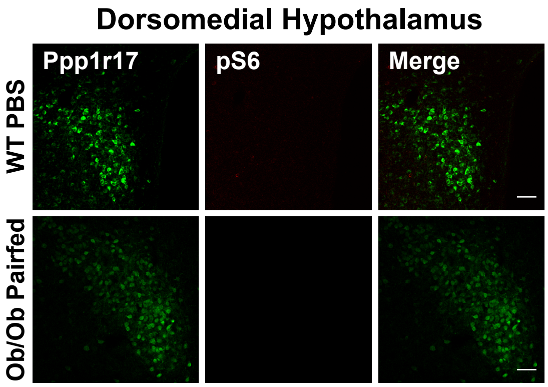

Figure S5. pS6 expression in DMH^{Ppp1r17} neurons in ob/ob mice is reduced by 14 day pair-feeding. ISH for Ppp1r17 and IHC for pS6 in the Dorsomedial Hypothalamus
